# CD8+ and CD4+ cytotoxic T cell escape mutations precede breakthrough SIVmac239 viremia in an elite controller

**DOI:** 10.1186/1742-4690-9-91

**Published:** 2012-11-06

**Authors:** Benjamin J Burwitz, Juan Pablo Giraldo-Vela, Jason Reed, Laura P Newman, Alexander T Bean, Francesca A Nimityongskul, Philip A Castrovinci, Nicholas J Maness, Enrique J Leon, Richard Rudersdorf, Jonah B Sacha

**Affiliations:** 1 Vaccine and Gene Therapy Institute, Oregon Health and Science University, 505 NW 185th, Beaverton, OR, 97006, USA; 2 Department of Pathology and Laboratory Medicine, University of Wisconsin, Madison, WI, 53706, USA; 3 Division of Microbiology, Tulane National Primate Research Center, Covington, LA, 70433, USA; 4 Division of Pathobiology and Immunology, Oregon National Primate Research Center, Oregon Health and Science University, Beaverton, OR, 97006, USA

**Keywords:** HIV, Cytolytic CD4+ T cells, Immune evasion

## Abstract

**Background:**

Virus-specific T cells are critical components in the containment of immunodeficiency virus infections. While the protective role of CD8+ T cells is well established by studies of CD8+ T cell-mediated viral escape, it remains unknown if CD4+ T cells can also impose sufficient selective pressure on replicating virus to drive the emergence of high-frequency escape variants. Identifying a high frequency CD4+ T cell driven escape mutation would provide compelling evidence of direct immunological pressure mediated by these cells.

**Results:**

Here, we studied a SIVmac239-infected elite controller rhesus macaque with a 1,000-fold spontaneous increase in plasma viral load that preceded disease progression and death from AIDS-related complications. We sequenced the viral genome pre- and post-breakthrough and demonstrate that CD8+ T cells drove the majority of the amino acid substitutions outside of Env. However, within a region of Gag p27^CA^ targeted only by CD4+ T cells, we identified a unique post-breakthrough mutation, Gag D205E, which abrogated CD4+ T cell recognition. Further, we demonstrate that the Gag p27^CA^-specific CD4+ T cells exhibited cytolytic activity and that SIV bearing the Gag D205E mutation escapes this CD4+ T cell effector function *ex vivo*.

**Conclusions:**

Cumulatively, these results confirm the importance of virus specific CD8+ T cells and demonstrate that CD4+ T cells can also exert significant selective pressure on immunodeficiency viruses *in vivo* during low-level viral replication. These results also suggest that further studies of CD4+ T cell escape should focus on cases of elite control with spontaneous viral breakthrough.

## Background

The critical role of CD4+ T cells in directing host immunity to chronic viral infections is well established. CD4+ T cells mediate a myriad of immune functions and are thus characterized into distinct T helper (Th) subsets such as Th1, Th2, Th17, and follicular Th cells depending on their specific effector activity [1]. In addition to coordinating adaptive immunity through their indirect helper functions, CD4+ T cells can also act directly to lyse virally infected cells (reviewed in reference [2]). Cytolytic CD4+ T cells are associated with control of chronic viral infections such as CMV [3], EBV [4], and, more recently, HIV-1 [5]. Furthermore, MHC-II-restricted killing [6] and CD4+ T cell selected escape mutations [7] have been observed *in vivo* in the LCMV mouse model of chronic viral infection.

Whether cytolytic CD4+ T cells exert similar selective pressure *in vivo* during chronic infection with immunodeficiency viruses like HIV and SIV remains unknown. During HIV infection, the protective role of HIV-specific CD4+ T cells is obfuscated by the preferential infection of these cells [8]. Nevertheless, HIV-specific CD4+ T cells correlate with control of viral replication in both HIV-infected patients [9] and SIV-infected rhesus macaques [10]. Furthermore, cytolytic CD4+ T cells capable of lysing infected macrophages vigorously expand during acute HIV infection [5] and establishment of elite control of SIV [11], suggesting these cells are involved in control of viremia. In agreement with these observations, experimental depletion of CD4+ T cells prior to SIV infection resulted in increased viral replication in macrophages and rapid progression to AIDS [12]. Finally, the modestly protective Env-based RV144 vaccine induced both non-neutralizing antibodies and cytolytic CD4+ T cells, raising the possibility that Env-specific CD4+ T cells contributed to the vaccine effect [13].

Despite cumulative data suggesting a protective effect of immunodeficiency virus-specific CD4+ T cells, their roles in containing viral replication and driving escape mutations remain controversial. Effective immune responses constrain HIV and SIV replication, resulting in the selection of viral escape variants as previously described for CD8+ T cells, neutralizing antibodies, and recently natural killer cells [14]. Previous studies suggest CD4+ T cells exert little to no selective pressure on HIV-1 [15-19] and that minor viral variants, which fail to stimulate CD4+ T cells, do not gain a replicative advantage within the viral quasispecies [15]. Therefore, identifying a high frequency CD4+ T cell driven escape mutation would provide compelling evidence of direct immunological pressure against HIV-1. To this end, we followed viral evolution in a unique SIVmac239-infected elite controller (<1,000 viral RNA copies/ml plasma) that experienced breakthrough viremia. We describe the emergence of both CD8+ and CD4+ T cell escape mutations, which preceded loss of SIVmac239 elite control. We demonstrate that Gag-specific CD4+ T cells capable of lysing infected macrophages no longer recognized the post-breakthrough mutant viral sequence, suggesting that CD4+ T cells drove the emergence of this escape variant *in vivo.* Selection of escape variants that evade immune surveillance and lead to breakthrough viremia is a well-described phenomenon for CD8+ T cells, such as described for the HLA-B*27-bound Gag KK10 epitope in HIV-infected patients [20,21]. However, to our knowledge, this is the first example of CD4+ T cells selecting for escape mutants in viral breakthrough.

## Results and discussion

### SIVmac239 breakthrough viremia in an elite controller macaque

Defining effective immune responses that constrain HIV/SIV replication is essential to the development of a prophylactic AIDS vaccine. Rare “elite controllers” (ECs) (HIV-1-infected humans with viral loads below 50 viral RNA (vRNA) copies/ml plasma and SIVmac239-infected rhesus macaques with viral loads below 1,000 vRNA copies/ml plasma in the absence of treatment) offer insight into the necessary components for protective immunity against AIDS viruses. Moreover, breakthrough viruses in ECs contain “footprints” of immune system pressure, yielding further discernment of particularly efficacious immune responses [20,21].

**Figure 1:**
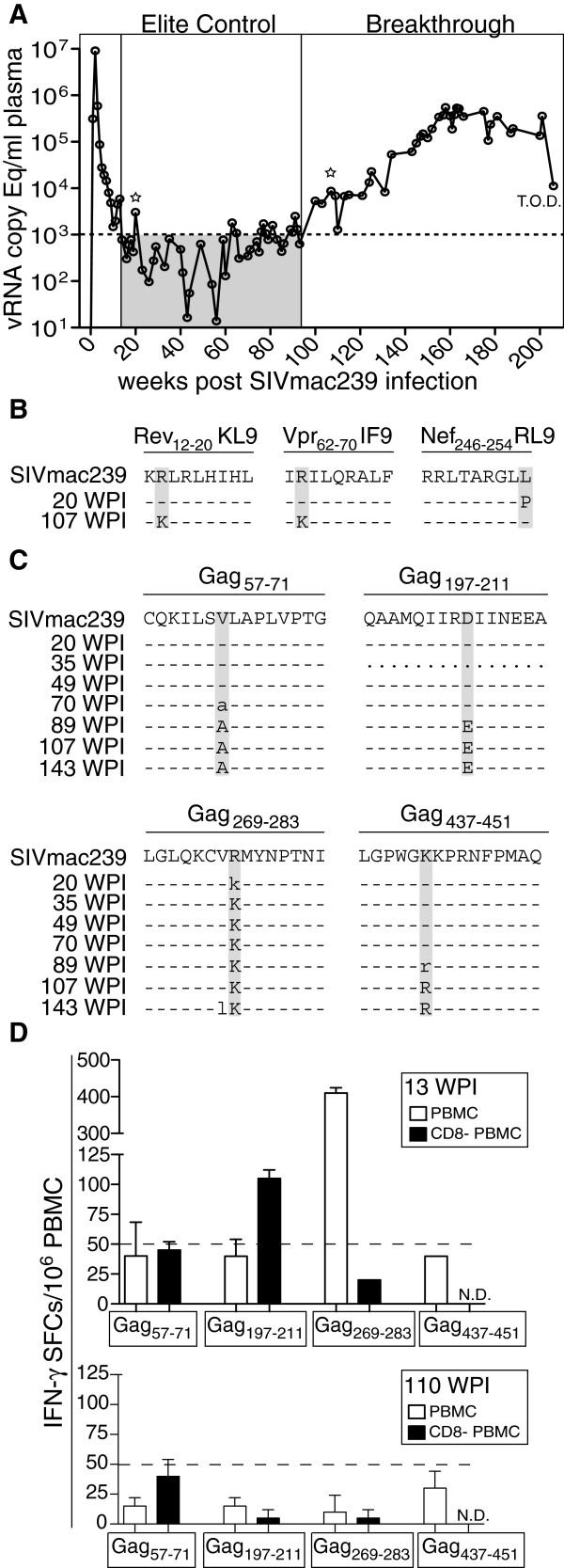
**SIVmac239 escape mutations in loss of SIV elite control. A**) SIVmac239 viral loads for animal r00032 post-infection. Dotted line indicates threshold for elite control. T.O.D. = Time of Death. Time of elite control and breakthrough are boxed. Stars indicate time of whole viral genome sequencing shown in Additional file 1: Figure S1. The limit of detection for viral load assay was 30 vRNA copies/ml plasma. **B**) The name, position, and sequence of SIVmac239 Mamu-B*008:01 epitopes exhibiting pre- and/or post-breakthrough variation are shown at 20 and 107 weeks post-infection (WPI) with mutations highlighted in grey. **C**) The name, position, and sequence of 4 regions in SIVmac239 Gag from animal r00032 exhibiting pre- and/or post-breakthrough variation are shown longitudinally with mutations highlighted in grey. Dashes indicate no change from wild type SIVmac239. Periods indicate no data. **D**) IFN-γ ELISpot measurements of immune responses against the four regions of SIVmac239 Gag shown in panel C; 13 weeks post-infection (top panel) and 110 weeks post-infection (bottom panel). White bars = PBMC effectors. Black bars = CD8+ T cell-depleted PBMC effectors. Dotted line represents limit of detection for the assay. N.D. = no data. Bars indicate the mean of duplicate wells with standard deviations shown.

In a cohort of >200 SIVmac239-infected rhesus macaques, we identified a Mamu-A1*002:01+ (formerly A*02)/Mamu-B*008:01+ (formerly B*08) animal, r00032, that maintained elite control (<1,000 copies vRNA/ml plasma) for over one year. A detailed study of this animal’s acute phase infection has previously been reported [22,23]. At ~96 weeks post-infection, the animal experienced spontaneous viral breakthrough, defined as the time point where viral load irreversibly crossed the elite control threshold of 1,000 copies vRNA/ml plasma (Figure 1A). We hypothesized that breakthrough virus harbored escape mutations that eluded effective immune surveillance. Therefore, we sequenced the entire viral genome during a spike in viremia early during elite control (20 weeks post infection) and immediately following viral breakthrough (107 weeks post infection) (Figure 1A). Analysis of pre- and post-breakthrough virus identified nineteen unique amino acid substitutions post-breakthrough with the majority (12/19 mutations) clustered in Gag and Env (Additional file 1: Figure S1). Two substitutions were escape mutations in the Mamu-B*008:01-bound Rev_12-20_KL9 and Vpr_62-70_IF9 CD8+ T cell subdominant epitopes while an escape mutation in the Mamu-B*008:01-bound Nef_246-254_RL9 epitope reverted to wild type SIVmac239 (Figure 1B). No other mutations occurred within defined Mamu-A1*002:01- or Mamu-B*008:01-bound epitopes, but early acute phase escape from immunodominant CD8+ T cells targeting the Mamu-B*008:01-bound Vif_123-131_RL9 and Vif_172-179_RL8 epitopes was maintained (Additional file 1: Figure S1).

All seven post-breakthrough substitutions in Env were common in a cohort of fifty-five chronically SIVmac239-infected animals (Additional file 2: Figure S2). In contrast, the four post-breakthrough substitutions in Gag were found only in r00032 (Additional file 3: Figure S3). Because Gag-specific CD8+ T cell responses correlate with lower viral loads [24] and escape from Gag-specific CD8+ T cells can lead to breakthrough viremia [20,21,25], we focused on these unique Gag substitutions present in breakthrough virus from r00032. We longitudinally sequenced these four regions and identified two changes, Gag R276K and V63A, which were first observed at 20 and 70 weeks post-infection, respectively (Figure 1C, left column). In contrast, mutations Gag D205E and K442R immediately preceded breakthrough viremia (Figure 1C, right column). Interestingly, the Gag D205E mutation in SIVmac239 has been shown to abrogate the protective effect of Gag_206-216_IL11-specific CD8+ T cells in *90-120-Ia* haplotype+ Burmese rhesus macaques [26], and also results in a loss of viral fitness [27]. We utilized *ex vivo* IFN-γ ELISpot to assess the immune responses targeting these regions of Gag. *Ex vivo* IFN-γ ELISpot analysis showed that only CD4+ T cells, and not CD8+ T cells, targeted Gag D205 in r00032 at 13 weeks post infection while CD8+ T cells robustly targeted Gag R276 (Figure 1D). CD8+ and CD4+ T cell responses against Gag V63 were equivocally positive, while we detected no responses against Gag K442 at this early time point. No IFN-γ CD8+ or CD4+ T cell responses were detected at 110 weeks post-infection, a post-breakthrough time point at which high-frequency substitutions were present at all four Gag positions (Figure 1C,D). These data suggested that both CD8+ and CD4+ T cells were driving viral evolution during elite control and that escape from these responses may have contributed to viral breakthrough.

### Both CD8+ and CD4+ T cells select for escape mutations

**Figure 2:**
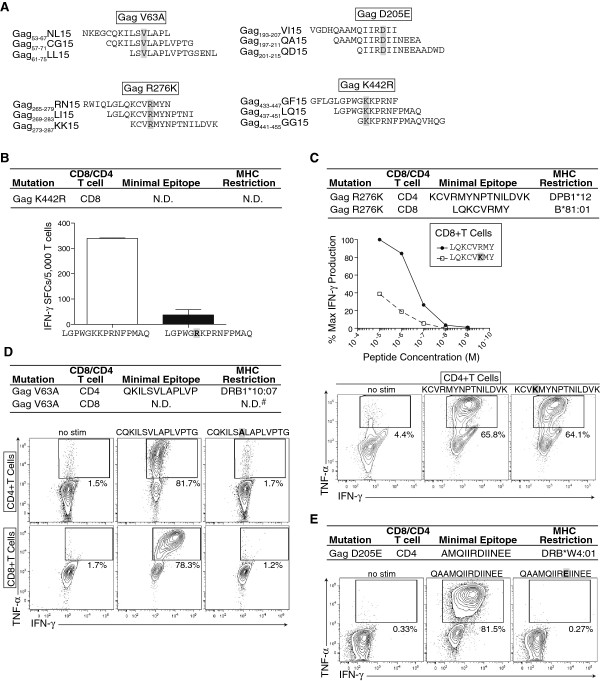
**Amino acid changes in SIV Gag from r00032 post breakthrough are CD8+ and CD4+ T cell escape mutations. A**) Schematic of the overlapping 15-mer peptides used to culture CD8+ and CD4+ T cell responses targeting the indicated regions of Gag, which exhibited pre- breakthrough substitutions. **B**) IFN-γ ELISpot measurements showing the response against SIVmac239 Gag_437-451_LQ15 and the mutant peptide containing Gag K442R by a CD8+ T cell line. White bar = Response against peptide Gag_437-451_LQ15. Black bar = Response against mutant peptide containing Gag K442R. Bars indicate the mean of duplicate wells with standard deviations shown. The point mutation is highlighted in grey and bold. **C**) IFN-γ ELISpot measurements showing the response against serial dilutions of SIVmac239 Gag_271-278_LY8 and the mutant peptide containing Gag R276K by a Mamu-B*81:01-restricted CD8+ T cell line (top panel). Data are normalized to percent maximum IFN-γ-production and are the mean of duplicate wells. Intracellular cytokine staining showing the response against no stimulation, SIVmac239 Gag_273-287_KK15, and the mutant peptide containing Gag R276K by a DRB1*12-restricted CD4+ T cell line (bottom panel). The point mutation is highlighted in grey and bold. **D**) Intracellular cytokine staining showing the response against no stimulation, SIVmac239 Gag_57-71_CG15, and the mutant peptide containing Gag V63A by a DRB1*10:07-restricted CD4+ T cell line (top row) and a CD8+ T cell line (bottom row) Mamu-A*02 or Mamu-B*008:01. The point mutation is highlighted in grey and bold. **E**) Intracellular cytokine staining showing the response against no stimulation, SIVmac239 Gag_197-211_QA15, and the mutant peptide containing Gag D205E by a DRB*W4:01-restricted CD4+ T cell line. The point mutation is highlighted in grey and bold. All data shown are representative of 3 or more independent experiments. N.D. = not determined. # = MHC-I restriction not Mamu-A1*002:01 or Mamu-B*008:01.

CD4+ T cells impose selective pressure and drive the emergence of high-frequency escape variants in RNA viruses like LCMV [7]. Despite this and the linkage of certain MHC-II molecules with lower HIV-1/SIV viral loads [10,28], CD4+ T cell escape is not thought to occur in immunodeficiency viruses due in part to preferential infection of virus-specific CD4+ T cells [15-19]. Furthermore, escape from CD8+ T cells contributes significantly to HIV/SIV sequence evolution outside of Env [29]. Therefore, it was possible that the observed post-breakthrough Gag substitutions were escape mutations driven by CD8+ T cell responses below our limit of *ex vivo* detection. To explore this possibility, we characterized the cellular immune responses against Gag by culturing r00032 PBMC *in vitro* with the overlapping 15-mer peptides corresponding to the four evolving regions (Figure 2A).

CD8+ T cells drove the emergence of the Gag K442R escape mutation as only Gag_437-451_LQ15-specific CD8+ T cells expanded in culture. Additionally, these CD8+ T cells failed to recognize the mutant sequence in an *in vitro* recognition assay (Figure 2B). Gag R276 was targeted by both Mamu-B*81:01-restricted Gag_271-278_LY8-specific CD8+ T cells and DPB1*12-restricted Gag_273-287_KK15-specific CD4+ T cells. However, the Mamu-B*81:01-restricted Gag_271-278_LY8-specific CD8+ T cells likely drove the Gag R276K mutation as these cells failed to recognize the post-breakthrough sequence while the DPB1*12-restricted Gag_273-287_KK15-specific CD4+ T cells efficiently responded to both pre- and post- breakthrough sequences (Figure 2C). Similarly, Gag V63 was targeted by both DRB1*10:07-restricted Gag_57-71_CG15-specific CD4+ T cells and Gag_57-71_CG15-specific CD8+ T cells of unknown MHC-I restriction. It is impossible to distinguish the relative importance of the CD8+ and CD4+ T cell responses in driving the Gag V63A substitution as it abrogated recognition by both the Gag_57-71_CG15-specific CD8+ and CD4+ T cells (Figure 2D). In contrast, Gag D205 was targeted solely by Gag_197-211_QA15-specific CD4+ T cells restricted by DRB*W4:01 (Figure 1D and Figure 2E). Repeated attempts to measure or isolate CD8+ T cells recognizing this region of Gag were unsuccessful, suggesting the Gag D205E substitution was solely CD4+ T cell driven. Accordingly, the Gag D205E substitution completely abrogated recognition by Gag_197-211_QA15-specific DRB*W4:01-restricted CD4+ T cells (Figure 2E). This finding, although unexpected, agrees with a recent report that CD4+ T cells are able to drive escape substitutions in HIV-1 *in vitro* (Philip Norris, personal communication).

### Characterization of CD4+ T cell SIV recognition and effector function

**Figure 3:**
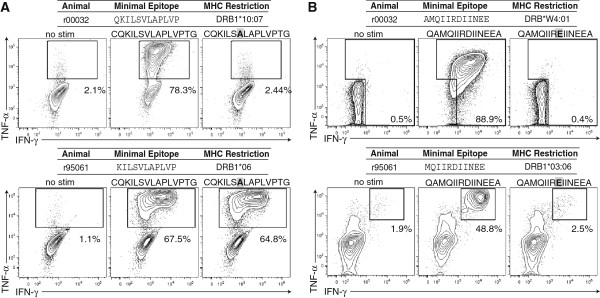
**SIV Gag D205E, but not V63A mutation abrogates recognition of CD4+ T cells restricted by alternate MHC-II molecules. A**) Comparison of recognition of wild type SIVmac239 Gag_57-71_CG15 and the mutant peptide containing the V63A point mutation or DRB1*06 (bottom panel). Flow data are shown as IFN-γ versus TNF-α, and percentages are cytokine-positive cells. **B**) Comparison of recognition of wild type SIVmac239 Gag_197-211_QA15 and Gag_197-211_QA15 bearing the D205E point mutation (highlighted in grey and bold) by Gag_197-211_QA15-specific CD4+ T cells restricted by either DRB*W4:01 (top panel) or DRB1*03:06 (bottom panel). All data shown are representative of 3 or more independent single replicate experiments.

CD4+ T cell epitopes are considered “promiscuous” due to their ability to bind multiple MHC-II molecules. In addition to this MHC-II binding promiscuity, it is believed that CD4+ T cells can tolerate more sequence diversity within their targeted epitope than CD8+ T cells. Therefore, we and others have suggested that promiscuous CD4+ T cell epitopes would be ideal targets for inclusion in an HIV-1 vaccine in order to minimize sequence divergence between the vaccine and circulating HIV strains [11,30,31]. Minimal data exist, however, on how viral variation affects recognition of CD4+ T cell epitopes presented on multiple MHC-II molecules. Therefore, we next explored what effect the Gag V63A and D205E breakthrough mutations would have on recognition by CD4+ T cells targeting this region, yet restricted by different MHC-II molecules. We have previously reported that CD4+ T cells specific for the Gag_57-71_CG15 and Gag_197-211_QA15 epitopes are alternately restricted by DRB1*06 and DRB1*03:06, respectively, and expand vigorously *in vivo* during reestablishment of elite control following CD8+ cell depletion [11]. We compared Gag_57-71_CG15- and Gag_197-211_QA15-specific CD4+ T cells from r00032 to those from r95061, an unrelated EC that expresses the alternate MHC-II restricting alleles DRB1*06 and DRB1*03:06. Although DRB1*10:07-restricted Gag_57-71_CG15-specific CD4+ T cells failed to recognize the Gag V63A mutation, DRB1*06-restricted CD4+ T cells specific for the same epitope recognized both the wild type and mutant sequences efficiently (Figure 3A). In contrast, the Gag D205E mutation ablated recognition by Gag_197-211_QA15-specific CD4+ T cells restricted by either DRB*W4:01 or DRB1*03:06 (Figure 3B). Therefore, while targeting promiscuous MHC-II-binding epitopes is an attractive strategic approach for maximizing population coverage for an HIV vaccine, even a single conservative amino acid substitution can abrogate recognition by CD4+ T cells restricted by different MHC-II molecules.

**Figure 4:**
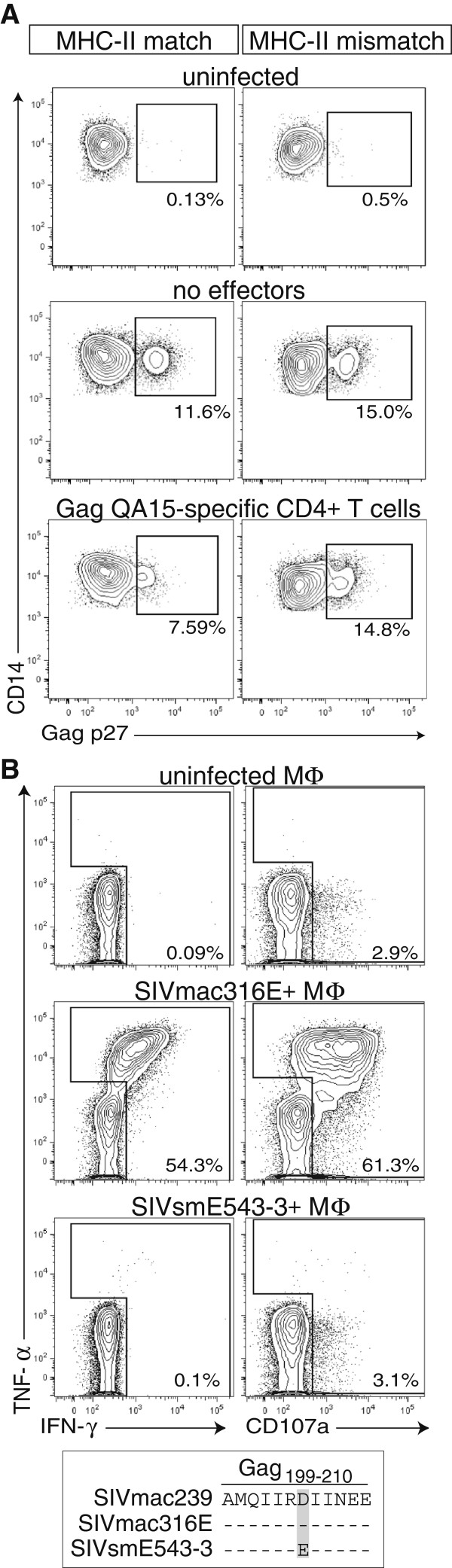
**SIV Gag D205E abrogates the effector function of Gag**_**197-211**_**QA15-specific CD4+ T cells. A**) DRB*W4:01-positive (MHC-II match) or -negative (MHC-II mismatch) macrophages were infected with SIVmac316E for 24 hours, then co-cultured with Gag_197-211_QA15-specific, DRB*W4:01-restricted CD4+ T cell clones at an E:T of 1:1 for 24 hours. Macrophages were then stained for surface expression of CD14 and intracellular SIV Gag p27. Percentages indicate Gag p27 staining. **B**) Recognition of uninfected, SIVmac316E-infected, or SIVsmE543-3-infected autologous macrophages by a DRB*W4:01-restricted, Gag_197-211_QA15-specific CD4+ T cell line. Flow data are shown as IFN-γ versus TNF-α or CD107a, and percentages are cytokine-positive cells. Lower box indicates sequence identity of core Gag_199-210_AE12 epitope in utilized SIV strains. All data shown are representative of 3 or more independent single replicate experiments.

Because it appeared that Gag_197-211_QA15-specific CD4+ T cells had selected for the Gag D205E escape mutation *in vivo,* we next explored if these cells could mediate direct effector function. We and others have previously demonstrated that CD4+ T cells contribute to elite control by directly lysing infected macrophages [5,11,32]. Therefore, we performed a previously described elimination assay that uses virally infected monocyte-derived macrophages as targets [11]. The Gag_197-211_QA15-specific CD4+ T cells mediated cytolytic activity and consistently eliminated ~30% of the infected macrophages in a MHC-II restricted manner (Figure 4A). Finally, we measured what effect virus bearing the Gag D205E mutation had on the ability of Gag_197-211_QA15-specific CD4+ T cells to degranulate (as measured by CD107a). In agreement with our elimination assay, DRB*W4:01-restricted Gag_197-211_QA15-specific CD4+ T cells efficiently degranulated in response to SIVmac316E-infected macrophages, a virus containing the wildtype Gag_197-211_QA15 sequence (Figure 4B). In contrast, these same CD4+ T cells did not degranulate in response to MHC-II-matched macrophages infected with SIVsmE543-3, a virus containing the Gag D205E mutation (Figure 4B). Therefore, viruses bearing the Gag D205E mutation were able to evade immune surveillance by Gag_197-211_QA15-specific CD4+ T cells.

### Gag_197-211_QA15-specific CD4+ T cells do not always select for Gag D250E escape variants

CD4+ T cells selected for the escape mutation Gag D205E in r00032. However, whether this was common in animals with CD4+ T cells targeting Gag_197-211_QA15 remained undetermined. We therefore identified two additional DRB*W4:01+ animals from our cohort of 55 animals. One animal, r95116, was infected with SIVsmE660 as part of a previously described SIV vaccine study [33]. The challenge virus SIVsmE660 contained the Gag D205E substitution, thus precluding the emergence of a Gag_197-211_QA15-specific CD4+ T cell response. The second animal, r04072, was infected with 8X-SIVmac239, a mutant of SIVmac239 that contains escape mutations with eight Mamu-B*008:01-bound CD8+ T cell epitopes [34]. Despite being Mamu-B*008:01+, r04072 maintained high viral loads throughout infection (Additional file 4: Figure S4A). We longitudinally sequenced virus from r04072 and observed no sequence variation in the Gag_197-211_QA15 epitope despite a cytotoxic Gag_197-211_QA15-specific CD4+ T cell response measured directly *ex vivo* by CD107a cell surface expression [35] (Additional file 4: Figure S4B,C). Although Gag_197-211_QA15-specific CD4+ T cells did not select for escape in r04072, this data further support our *in vitro* results showing cytolytic activity by Gag_197-211_QA15-specific CD4+ T cells. The data are also in line with recent reports of *ex vivo* cytolytic CD4+ T cell responses against HIV Gag [2,36]. Finally, this data suggest that CD4+ T cell escape may only occur in rare cases, such as in ECs with low-level viral replication followed by sudden breakthrough viremia. We therefore also sequenced virus from six ECs, which underwent experimental CD8+ cell depletion, leading to a rapid spike in viremia [11,37]. These animals all expressed either DRB1*03:06 (the alternate allele restricting Gag_197-211_QA15-specific CD4+ T cell responses), or the alleles DRB1*06 and/or DRB1*10:07 (which restrict Gag_57-71_CG15-specific CD4+ T cells) (Additional file 5: Figure S5A). Sequences were acquired at the peak of viral replication following CD8+ cell depletion and following the resolution of viremia after the repopulation of CD8+ cells. We did not observe Gag V63A or D205E escape mutations in any of the animals at either of the time points (Additional file 5: Figure S5B).

The lack of the Gag D205E escape mutation in other SIV infected rhesus macaques expressing DRB*W4:01 or DRB1*03:06 indicates this mutation may be rare and associated with natural spontaneous viral breakthrough in ECs. Thus, we conclude that ECs with viral breakthrough likely provide the best model for the study of CD4+ T cell driven viral escape. Given the paucity of SIV infected rhesus macaques that have been MHC-II typed and shown to express DRB*W4:01 or DRB1*03:06, the phenomenon of CD4+ T cell driven Gag D205E escape will require a larger cohort of infected animals with more extensive MHC-II typing.

CD8+ T cells, neutralizing antibodies, and antiretroviral drugs all drive HIV sequence variation. Here, we provide the first evidence that CD4+ T cells can also drive viral variation. However, we cannot dismiss the possibility that other immune mechanisms may play a role in the escape observed within the CD4+ T cell epitope Gag_197-211_QA15. For example, a recent study by Chung *et. al.* indicates that antibody-dependent cellular cytotoxicity (ADCC) can drive HIV escape mutations in Env [38]. ADCC epitopes have been identified in internal proteins including Vpu and Pol, but were not found in Gag in a cohort of 26 HIV-infected individuals [39]. Nonetheless, immune pressure from an unidentified non-CD4+ T cell source could conceivably be responsible for the Gag D205E mutation. Interestingly, Gag_197-211_QA15 is located in a conserved region of Gag that has been proposed as an ideal target for T cell-based vaccines, and position Gag 205 seems to be under selective pressure as both Gag D205 and Gag E205 are found in HIV clade A/C and B/D consensus sequences, respectively [40].

Breakthrough viremia in r00032 was temporally associated with the mutations Gag V63A and D205E, implying that increased viral replication was a consequence of immune escape. However, CD4+ T cell dysfunction and exhaustion during chronic HIV and SIV infection are positively correlated with viral load and may also have played a role in breakthrough viremia. Future studies of CD4+ T cell escape in HIV and SIV ECs should focus on the interplay between viral replication, viral variation, and CD4+ T cell dysfunction.

Elite control and long-term non-progression have been associated with CD8+ T cell responses, CD4+ T cell responses, and NK cells [9,10,41-45]. Therefore, the CD4+ T cell selected mutation we observed prior to breakthrough viremia is likely only one of many complex determinants in loss of elite control. Indeed, we observed multiple CD8+ T cell selected escape mutations emerging immediately prior to viral breakthrough; thus the contribution of CD8+ and CD4+ T cell responses to viral breakthrough in r00032 requires an assessment of viral variant fitness outside the scope of this study. Nevertheless, the CD4+ T cell-driven mutation at Gag D205E represents the first description of a high-frequency escape mutation in immunodeficiency virus infection and further supports the growing evidence implicating CD4+ T cells as direct suppressors of viral replication.

## Conclusions

Uncertainty remains about the importance of CD4+ T cells as effectors against HIV-1, given that expansion of HIV-specific CD4+ T cells may provide additional targets for viral replication [8]. In contrast with other studies [15-19,46], we show here that CD4+ T cells exert strong immune pressure *in vivo* in the setting of limited viral replication*,* evidenced by the emergence of a high frequency CD4+ T cell escape mutation during elite control. Taken together, these data indicate that elicitation of cytolytic CD4+ T cells should be considered in future vaccines against HIV.

## Methods

### Animals

Rhesus macaques at the Wisconsin National Primate Research Center were cared for by regulations set by the Guide for Care and Use of Laboratory Animals of the National Research Council and the University of Wisconsin Institutional Animal Care and Use Committee. The infection of r00032 was described previously [23].

### Sequencing and quantification of viral RNA in plasma

Cell-free plasma was obtained from EDTA anti-coagulated whole blood by using Ficoll-Paque Plus (GE Healthcare Bioscience) and density centrifugation. Viral RNA in the plasma was isolated by using the QIAamp MinElute virus spin kit (Qiagen, Valencia, CA) according to the manufacturer’s instructions. Viral RNA was reverse transcribed and amplified using the One-Step reverse transcription-PCR kit (Qiagen), generating amplicons spanning SIVmac239. Bulk Sanger sequencing was performed on an ABI 3730xl DNA analyzer using ET terminator technology (GE Healthcare). Sequences were assembled against the SIVmac239 reference sequence (GenBank accession M33262) and conceptually translated using CodonCode Aligner version 3.7.1.1 (CodonCode Corporation) and MacVector 11.1 trial version (MacVector,Inc). Plasma SIVmac239 concentrations were monitored by quantitative reverse transcription-PCR as previously described [23]. The limit of detection for this assay is 30 vRNA eq/ml.

### IFN-γ ELISpot

Frozen PBMC were used in ELISpot assays for the detection of IFN-γ–secreting cells as previously described [10]. For measuring CD4+ T cell responses, we depleted PBMC of CD8+ cells using a CD8-Microbead kit for nonhuman primates (Miltenyi Biotec) according to manufacturer’s instructions prior to performing the ELISpot assay. Responses were considered positive if the mean number of spot-forming cells (SFC) of duplicate sample wells exceed background plus two standard deviations. Responses of less than 50 SFC per million PBMC were considered negative (below the limit of detection). For IFN-γ ELISpot assays using *in vitro* expanded cell culture, 5,000 T cells were incubated with 20,000 autologous BLCL and the peptide of interest in each well. Positive responses were determined using a one-tailed t test and an alpha level of 0.05, where the null hypothesis was that the background level would be greater than or equal to the treatment level. If determined to be positive statistically, the values were reported as the average of the test wells minus the average of all negative-control wells. Peptides used in these assays were obtained through the AIDS Research and Reference Reagent Program, Division of AIDS, NIAID, NIH.

### Intracellular cytokine staining (ICS)

ICS was performed as described previously [10]. Briefly, 1 × 10^5^ effector cells were incubated with peptide-pulsed autologous B cells or infected macrophages in the presence of CD107a antibody for 90 min followed by the addition of 5 μg/mL brefeldin A and Golgistop followed by an additional 5 hour incubation. Cells were subsequently stained for CD4 and CD8 surface expression followed by intracellular CD3, IFN-γ, and TNF-α, fixed in 2% PFA, acquired on a BD-LSRII flow cytometer, and analyzed with FlowJo.

### T cell and macrophage in vitro culture

T-cell *in vitro* cultures were initiated with freshly isolated PBMC from animal r00032 co-cultured with irradiated, autologous BLCL pulsed with the three overlapping SIVmac239 15-mer peptides of interest. Following two weekly restimulations, limiting dilution was performed and individual clones were selected from the highest dilutions showing expansion. Cells were maintained throughout *in vitro* culture in R15-100 media (RPMI media + 15% FCS, supplemented with 100 IU rhIL-2). Specificity was examined in both the bulk T cell line and T cell clones generated by limiting dilution by using each 15-mer peptide individually, followed by truncated versions of the 15-mer peptide as appropriate. To generate macrophages, CD14^+^ monocytes were isolated via MACS using CD14 microbeads (Miltenyi Biotec) and incubated in 50% fresh R10 media (RPMI + 10% FCS) and 50% KPB-M15-conditioned R10 media supplemented with 10 ng/mL M-CSF (Sigma) for 6 days, feeding every other day. Macropahges were consistently >95% CD14^+^ and were infected with 100 ng of Gag p27 SIVmac316E (kindly provided by Ronald Desrosiers) or SIVsmE543-3 (kindly provided by Vanessa Hirsch) by spinoculation.

### Macrophage 24 hour elimination assay

Macrophages (5 × 10^4^) were infected as described above, the infection was allowed to proceed for 24 h, and they were then cocultured with SIV-specific CD4+ T cells at an E:T of 1:1 at 37°C for 24 h. The culture was then surface stained with antibodies against CD4, CD14, and HLA-DR (BD Biosciences), followed by intracellular staining for Gag p27 using Fix and Perm (Caltag Laboratories) and the 55–2 F12 Gag p27 antibody (National Institutes of Health AIDS Reagent Database) at ≈ 0.7 mg/mL. Events were collected on a BD FACSCaliber and analyzed using FlowJo software. T cell cells were gated out using forward and side scatter dot plots and excluding CD3+ events.

### Mapping of MHC restriction and minimal epitope

A cDNA library of r00032 MHC-I and –II alleles was generated as previously described [10]. Either .221 (MHC-I-devoid) or RM3 (MHC-II-devoid) cells were transfected with each allele, pulsed with the peptide of interest, and then used as antigen presenting cells to T cells. Truncated versions of the 15-mer peptide used to culture cell lines were pulsed onto autologous BLCL and used in ICS to determine the minimal epitope by identifying the minimal peptide that resulted in maximal cytokine secretion.

## Competing interests

The authors declare that they have no competing interests.

## Authors’ contributions

Designed experiments: JPG, JBS. Performed experiments: BJB, JPG, JR, LPN, ATB, FAN, PAC, NJM, EJL, RR, JBS. Analyzed data: BJB, JPG, NJM, EJL, JBS. Wrote manuscript: BJB, JBS. All authors read and approved the final manuscript.

## Supplementary Material

Additional file 1 **Figure S1.** Sequence of plasma SIV from animal r00032. Bulk Sanger sequence of plasma SIV from r00032 at 20 weeks post infection (WPI) and 107 WPI for **a**) Gag, **b**) Vif, **c**) Vpx, **d**) Vpr, **e**) Tat, **f**) Rev, **g**) Nef, **h**) Env, and **i**) Pol. Grey boxes indicate positions of variation present at 107 WPI that were absent at 20 WPI. Yellow boxes indicate previously described CD8+ T cell responses restricted by Mamu-A1*002:01. Blue boxes indicate previously described CD8+ T cell responses restricted by Mamu-B*008:01. Light red boxes indicate the variable loops of SIVmac239 Env. A novel CD8+ T cell response (B*80:01 Gag _365-372_ AL8) is boxed in Gag. Click here for file

Additional file 2 **Figure S2.** Amino acid changes in SIV Env from r00032 post breakthrough are common mutations. Bulk Sanger sequence comparing SIVmac239 Env mutations found in r00032 at 107 WPI to mutations found in a cohort of 55 SIVmac239-infected rhesus macaques from the Wisconsin National Primate Research Center during chronic SIVmac239 infection or at time of death. Grey boxes indicate positions of variation found in viral sequence from r00032 with respect to the SIVmac239 reference sequence. Colons indicate regions without SIVmac239 sequence. Click here for file

Additional file 3 **Figure S3.** Amino acid changes in SIV Gag from r00032 post breakthrough are unique mutations. Bulk Sanger sequence comparing SIVmac239 Gag mutations found in r00032 at 107 WPI to mutations found in a cohort of 55 SIVmac239-infected rhesus macaques from the Wisconsin National Primate Research Center during chronic SIVmac239 infection or at time of death. Grey boxes indicate positions of variation found in viral sequence from r00032 with respect to the SIVmac239 reference sequence. Colons indicate regions without SIVmac239 sequence. Click here for file

Additional file 4 **Figure S4.** A DRB*W4:01+ animal with unresolved 8X-SIVmac239 replication does not select for escape within the Gag_199-210_AE12 epitope despite the presence of Gag_199-210_AE12-specific cytolytic CD4+ T cells. (**A**) The SIVmac239 viral loads for animal r04072 post-infection. This Mamu-B*008:01+ animal was infected with a mutant SIVmac239 virus, which contained escape mutations within eight Mamu-B*008:01 CD8+ T cell epitopes as described previously [34]. T.O.D. = Time of Death. (**B**) The longitudinal sequence of SIVmac239 Gag_197-211_ QA15 from animal r04072 with position 205 highlighted in grey. (**C**) Direct *ex vivo* analysis of the ability of CD4+ T cells from r04072 to degranulate (as measured by CD107a) in response to whole AT-2-inactivated SIVmac239 or the Gag_197-211_QA15 peptide. Data is representative of two independent single replicate experiments performed with PBMC samples from 100 WPI. Click here for file

Additional file 5 **Figure S5.** CD8-depleted SIV ECs show no evidence of CD4+ T cell mediated escape within two highly targeted Gag CD4+ T cell epitopes. (**A**) SIV ECs from a previously described CD8+ cell depletion experiment [11,37] are listed along with their MHC-II molecules known to target Gag_57-71_ CG15 and Gag_197-211_ QA15. (**B**) Sequence of Gag_57-71_ CG15 and Gag_197-211_ QA15 at 14 days (peak) and >28 days (post) post experimental CD8 depletion. No variation is observed within these two regions of Gag. Click here for file
