# Supplementary material for: CD8+ and CD4+ cytotoxic T cell escape mutations precede breakthrough SIVmac239 viremia in an elite controller
Source: Retrovirology. 2012 Nov 6;9:91. doi: 10.1186/1742-4690-9-91 (PMC3496649; doi:10.1186/1742-4690-9-91)
Supplement: Additional file 1 — Figure S1. Sequence of plasma SIV from animal r00032. Bulk Sanger sequence of plasma SIV from r00032 at 20 weeks post infection (WPI) and 107 WPI for a) Gag, b) Vif, c) Vpx, d) Vpr, e) Tat, f) Rev, g) Nef, h) Env, and i) Pol. Grey boxes indicate positions of variation present at 107 WPI that were absent at 20 WPI. Yellow boxes indicate previously described CD8+ T cell responses restricted by Mamu-A1*002:01. Blue boxes indicate previously described CD8+ T cell responses restricted by Mamu-B*008:01. Light red boxes indicate the variable loops of SIVmac239 Env. A novel CD8+ T cell response (B*80:01 Gag 365-372 AL8) is boxed in Gag. [file 1742-4690-9-91-S1.pdf]

Supplemental Figure 1: Sequence of plasma SIV from animal r00032.

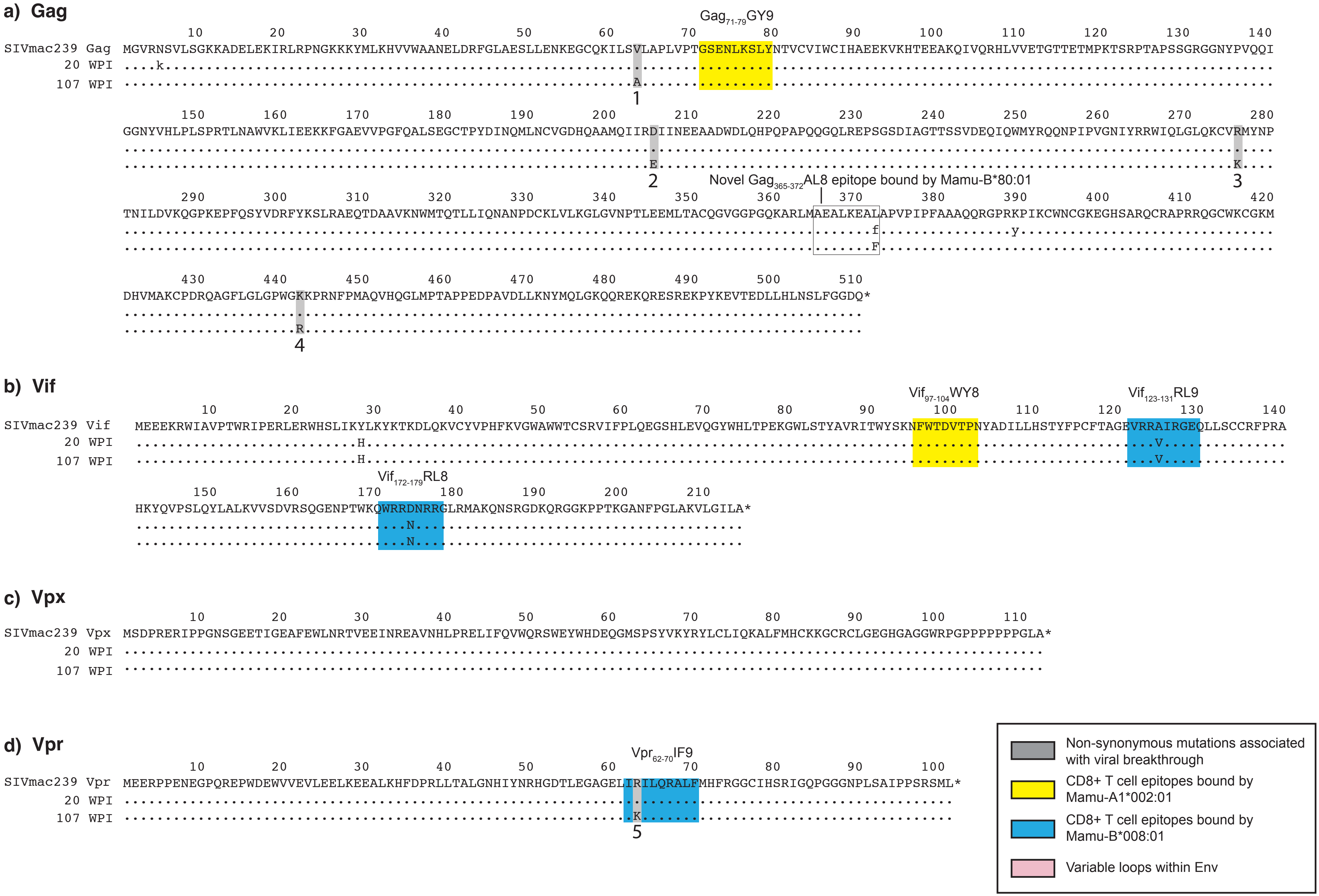

Supplemental Figure 1: Sequence of plasma SIV from animal r00032.

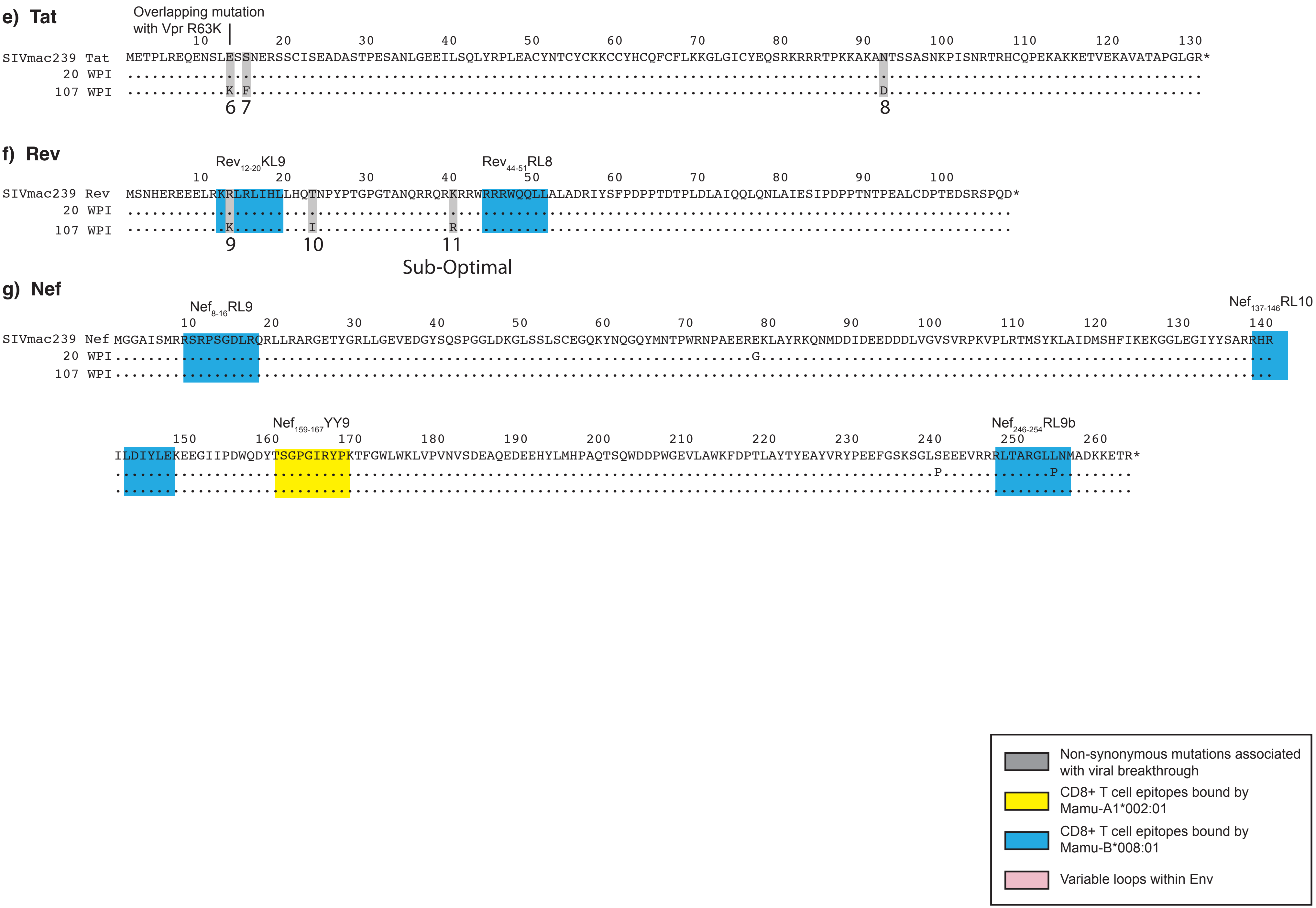

Supplemental Figure 1: Sequence of plasma SIV from animal r00032.

h) Env

```
                                                                                                                                  Variable Loop 1
                       10        20        30        40        50        60        70        80        90       100       110       120       130       140
SIVmac239 Env  MGCLGNQLLIAILLLSVYGIYCTLYVTVFYGVPAWRNATIPLFCATKNRDTWGTTQCLPDNGDYSEVALNVTESFDAWNNTVTEQAIEDVWQLFETSIKPCVKLSPLCITMRCNKSETDRWGLTKSITTTASTTSTTASA
       20 WPI  ............................................................................................................................................
      107 WPI  ..................................................................M................................................N.....................V..
                                                                                  12                                               13                    14

                                                                    Variable Loop 2
                      150       160       170       180       190       200       210       220       230       240       250       260       270       280
               KVDMVNETSSCIAQDNCTGLEQEQMISCKFNMTGLKRDKKKEYNETWYSADLVCEQGNNTGNESRCYMNHCNTSVIQESCDKHYWDAIRFRYCAPPGYALLRCNDTNYSGFMPKCSKVVVSSCTRMMETQTSTWFGFNGT
               ............................................................................................................................................
               ............................................................................................................................................

                                                         Variable Loop 3                                                                   Variable Loop 4
                      290       300       310       320       330       340       350       360       370       380       390       400       410       420
               RAENRTYIYWHGRDNRTIISLNKYYNLTMKCRRPGNKTVLPVTIMSGLVFHSQPINDRPKQAWCWFGGKWKDAIKEVKQTIVKHPRYTGTNNTDKINLTAPGGGDPEVTFMWTNCRGEFLYCKMNWFLNWVEDRNTANQK
               ............................................................................................................................................
               ........................................................................................................................................T...
                                                                                                                                                      15

                                                                    Variable Loop 5
                      430       440       450       460       470       480       490       500       510       520       530       540       550       560
               PKEQHKRNYVPCHIRQIINTWHKVGKNVYLPPREGDLTCNSTVTSLIANIDWIDGNQTNITMSAEVAELYRLELGDYKLVEITPIGLAPTDVKRYTTGGTSRNKRGVFVLGFLGFLATAGSAMGAASLTLTAQSRTLLAG
               ............................................................................................................................................
               ..........................................................................................G....A............................................
                                                                                                         16   17

                      570       580       590       600       610       620       630       640       650       660       670       680       690       700
               IVQQQQQLLDVVKRQQELLRLTVWGTKNLQTRVTAIEKYLKDQAQLNAWGCAFRQVCHTTVPWPNASLTPKWNNETWQEWERKVDFLEENITALLEEAQIQQEKNMYELQKLNSWDVFGNWFDLASWIKYIQYGVYIVVG
               ............................................................................................................................................
               ..................................................................N.........................................................................
                                                                                  18

                                                                                                         Env788-795RY8
                      710       720       730       740       750       760       770       780       790       800       810       820       830       840
               VILLRIVIYIVQMLAKLRQGYRPVFSSPPSYFQQTHIQQDPALPTREGKERDGGEGGGNSSWPWQIEYIHFLIRQLIRLLTWLFSNCRTLLSRVYQILQPILQRLSATLQRIREVLRTELTYLQYGWSYFHEAVQAVWRS
               ............................................................................................................................................
               ..................................................G.........................................................................................
                                                                  19

                      850       860       870       880
               ATETLAGAWGDLWETLRRGGRWILAIPRRIRQGLELTLL*
               .......................................
               .......................................
```

Grey	Non-synonymous mutations associated with viral breakthrough
Yellow	CD8+ T cell epitopes bound by Mamu-A1*002:01
Blue	CD8+ T cell epitopes bound by Mamu-B*008:01
Pink	Variable loops within Env

Supplemental Figure 1: Sequence of plasma SIV from animal r00032.

i) Pol

```
                      10        20        30        40        50        60        70        80        90       100       110       120       130       140
SIVmac239 Pol VLELWERGTLCKAMQSPKKTGMLEMWKNGPCYGQMPRQTGGFFRPWSMGKEAPQFPHGSSASGADANCSPRGPSCGSAKELHAVGQAAERKAERKQREALQGGDRGFAAPQFSLWRRPVVTAHIEGQPVEVLLDTGADDS
       20 WPI ............................................................................................................................................
      107 WPI ............................................................................................................................................

                     150       160       170       180       190       200       210       220       230       240       250       260       270       280
              IVTGIELGPHYTPKIVGGIGGFINTKEYKNVEIEVLGKRIKGTIMTGDTPINIFGRNLLTALGMSLNFPIAKVEPVKVALKPGKDGPKLKQWPLSKEKIVALREICEKMEKDGQLEEAPPTNPYNTPTFAIKKKDKNKWR
              ............................................................................................................................................
              .........r.........................................................l........................................................................
                      20                                                        21

                     290       300       310       320       330       340       350       360       370       380       390       400       410       420
              MLIDFRELNRVTQDFTEVQLGIPHPAGLAKRKRITVLDIGDAYFSIPLDEEFRQYTAFTLPSVNNAEPGKRYIYKVLPQGWKGSPAIFQYTMRHVLEPFRKANPDVTLVQYMDDILIASDRTDLEHDRVVLQSKELLNSI
              ............................................................................................................................................
              .............................................................................................r..............................................
                                                                                                            22

                     430       440       450       460       470       480       490       500       510       520       530       540       550       560
              GFSTPEEKFQKDPPFQWMGYELWPTKWKLQKIELPQRETWTVNDIQKLVGVLNWAAQIYPGIKTKHLCRLIRGKMTLTEEVQWTEMAEAEYEENKIILSQEQEGCYYQEGKPLEATVIKSQDNQWSYKIHQEDKILKVGK
              ............................................................................................................................................
              ............................................................................................................................................

                     570       580       590       600       610       620       630       640       650       660       670       680       690       700
              FAKIKNTHTNGVRLLAHVIQKIGKEAIVIWGQVPKFHLPVEKDVWEQWWTDYWQVTWIPEWDFISTPPLVRLVFNLVKDPIEGEETYYTDGSCNKQSKEGKAGYITDRGKDKVKVLEQTTNQQAELEAFLMALTDSGPKA
              ............................................................................................................................................
              ............................................................................................................................................

                     710       720       730       740       750       760       770       780       790       800       810       820       830       840
              NIIVDSQYVMGIITGCPTESESRLVNQIIEEMIKKSEIYVAWVPAHKGIGGNQEIDHLVSQGIRQVLFLEKIEPAQEEHDKYHSNVKELVFKFGLPRIVARQIVDTCDKCHQKGEAIHGQANSDLGTWQMDCTHLEGKII
              ............................................................................................................................................
              ............................................................................................................................................

                     850       860       870       880       890       900       910       920       930       940       950       960       970       980
              IVAVHVASGFIEAEVIPQETGRQTALFLLKLAGRWPITHLHTDNGANFASQEVKMVAWWAGIEHTFGVPYNPQSQGVVEAMNHHLKNQIDRIREQANSVETIVLMAVHCMNFKRRGGIGDMTPAERLINMITTEQEIQFQ
              ............................................................................................................................................
              ............................................................................................................................................

                     990      1000      1010      1020      1030      1040      1050      1060
              QSKNSKFKNFRVYYREGRDQLWKGPGELLWKGEGAVILKVGTDIKVVPRRKAKIIKDYGGGKEVDSSSHMEDTGEAREVA*
              ................................................................................
              ................................................................................
```

Non-synonymous mutations associated with viral breakthrough

CD8+ T cell epitopes bound by Mamu-A1*002:01

CD8+ T cell epitopes bound by Mamu-B*008:01

Variable loops within Env
