# Supplementary material for: CD8+ and CD4+ cytotoxic T cell escape mutations precede breakthrough SIVmac239 viremia in an elite controller
Source: Retrovirology. 2012 Nov 6;9:91. doi: 10.1186/1742-4690-9-91 (PMC3496649; doi:10.1186/1742-4690-9-91)
Supplement: Additional file 3 — Figure S3. Amino acid changes in SIV Gag from r00032 post breakthrough are unique mutations. Bulk Sanger sequence comparing SIVmac239 Gag mutations found in r00032 at 107 WPI to mutations found in a cohort of 55 SIVmac239-infected rhesus macaques from the Wisconsin National Primate Research Center during chronic SIVmac239 infection or at time of death. Grey boxes indicate positions of variation found in viral sequence from r00032 with respect to the SIVmac239 reference sequence. Colons indicate regions without SIVmac239 sequence. [file 1742-4690-9-91-S3.pdf]

Supplemental Figure 3: Amino acid changes in SIV Gag from r00032 post breakthrough are unique mutations.

Animal	Time Point	Gag_{57-71}	$Gag_{197-211}$	$Gag_{271-287}$	$Gag_{437-451}$
		CQKILSVLAPLVPTG	QAAMQIIRDIINEEA	LQKCVRMYNPTNILDVK	LGPWGKKPRNFPMAQ
r00032	107 WPI	------A--------	--------E------	-----K-----------	-----R---------
r00014	79 WPI	---------------	---------------	-----------------	:::::::::::::::
r00021	72 WPI	-----------M---	---------------	:::::::::::::::::	---------------
r00041	18 WPI	---------------	---------------	-----------------	:::::::::::::::
r00044	98 WPI	---------------	---------V-----	-----------------	---------------
r00045	197 WPI	-----------A---	---------------	-----------------	---------------
r00060	96 WPI	:::::::::::::::	---------V-----	:::::::::::::::::	---------------
r01064	129 WPI	---------------	---------------	:::::::::::::::::	---------------
r01088	49 WPI	---------------	---------------	-----------------	---------------
r80035	32 WPI	---------------	---------------	-----------------	---------------
r95096	480 WPI	-----------M---	---------V-----	---------A-------	---------------
r96107	62 WPI	---------------	---------------	-----------------	---------------
r96112	127 WPI	---------------	---------------	---------------I-	---------------
r97035	237 WPI	---------------	---------V-----	-----------------	---------------
r97073	88 WPI	:::::::::::::::	:::::::::::::::	-----------------	---------------
r97113	257 WPI	---------------	:::::::::::::::	-----------------	:::::::::::::::
r98030	65 WPI	---------------	---------------	-----------------	---------------
r99005	37 WPI	---------------	---------------	-----------------	---------------
rh2122	102 WPI	:::::::::::::::	---------V-----	-----------------	---------------
rh2161	43 WPI	---------------	:::::::::::::::	:::::::::::::::::	---------------
r80025	(TOD)	-----------x---	---------------	-----------------	---------------
r90131	(TOD)	---------------	---------------	-----------------	---------------
r87108	(TOD)	---------------	---------------	-----------------	---------------
r96114	(TOD)	---------------	---------------	-----------------	---------------
r95112	(TOD)	---------------	---------------	-----------------	---------------
r96081	(TOD)	---------------	---------------	-----------------	---------------
r97074	(TOD)	---------------	---------------	-----------------	---------------
r97009	(TOD)	---------------	---------------	-----------------	---------------
r81035	(TOD)	-----------m---	---------------	-----------------	---------------
r92077	(TOD)	---------------	---------------	-----------------	---------------
r87082	(TOD)	---------------	---------------	-----------------	---------------
r92050	(TOD)	---------------	---------------	-----------------	---------------
r96016	(TOD)	---------------	---------------	-----------------	---------------
r96135	(TOD)	---------------	---------------	-----------------	---------------
r95086	(TOD)	---------------	---------------	-----------------	---------------
r93062	(TOD)	---------------	---------------	-----------------	---------------
r80035	(TOD)	---------------	---------------	-----------------	---------------
r96123	(TOD)	---------------	---------------	-----------------	---------------
r95045	(TOD)	-----------m---	---------------	-----------------	---------------
r96020	(TOD)	---------------	---------------	-----------------	---------------
r85013	(TOD)	-----------m---	---------V-----	-----------------	---------------
r96104	(TOD)	---------------	---------------	-----------------	---------------
r96093	(TOD)	-----------M---	---------------	-----------------	---------------
r95084	(TOD)	-----------L---	---------V-----	-----------------	---------------
r96072	(TOD)	-----------L---	---------------	-----------------	---------------
r93057	(TOD)	---------------	---------V-----	-----------------	---------------
rh2127	(TOD)	-----------m---	---------------	-----------------	---------------
r95003	(TOD)	-----------M---	---------------	-----------------	---------------
rh2065	(TOD)	-----------m---	---------------	-----------------	---------------
r95058	(TOD)	-----------m---	---------v-----	-----------------	---------------
r95115	(TOD)	-----------m---	---------------	-----------------	---------------
rh1975	(TOD)	-----------M---	---------V-----	-----------------	---------------
r96118	(TOD)	-----------M---	---------V-----	-----------------	---------------
r96031	(TOD)	-----------l---	---------------	-----------------	---------------
rh1937	(TOD)	---V-------M---	---------V-----	-----------------	i--------------
Totals		------X--------	--------X------	-----X-----------	-----X---------
		(1/55)	(1/55)	(1/55)	(1/55)
