# Supplementary figures and images for: CD8+ and CD4+ cytotoxic T cell escape mutations precede breakthrough SIVmac239 viremia in an elite controller
Source: Retrovirology. 2012 Nov 6;9:91. doi: 10.1186/1742-4690-9-91 (PMC3496649; doi:10.1186/1742-4690-9-91)

Supplemental Figure 4

A

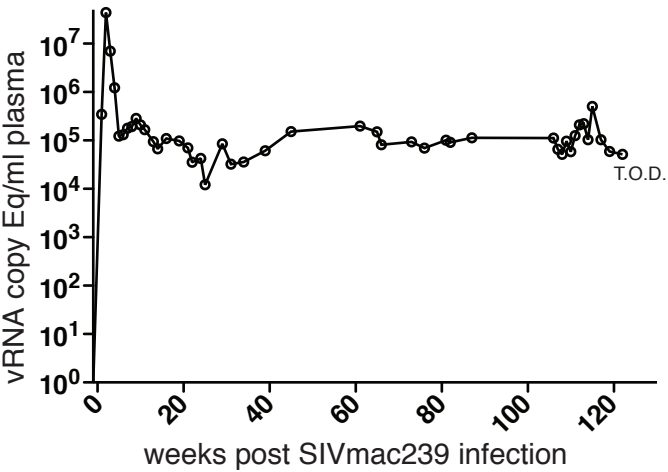

B

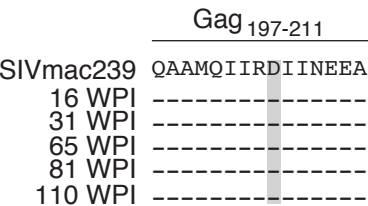

C

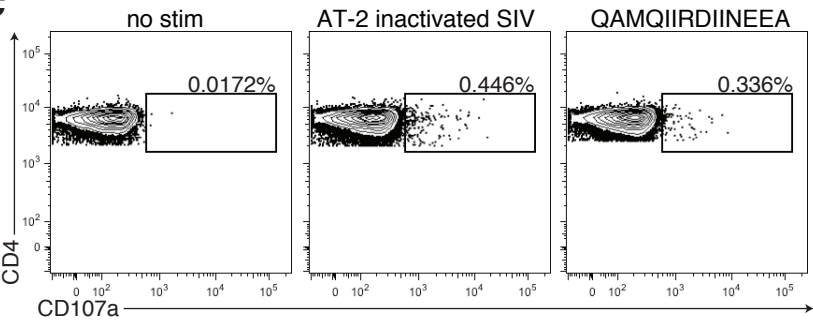

Supplement: Additional file 4 — Figure S4. A DRB*W4:01+ animal with unresolved 8X-SIVmac239 replication does not select for escape within the Gag199-210AE12 epitope despite the presence of Gag199-210AE12-specific cytolytic CD4+ T cells. (A) The SIVmac239 viral loads for animal r04072 post-infection. This Mamu-B*008:01+ animal was infected with a mutant SIVmac239 virus, which contained escape mutations within eight Mamu-B*008:01 CD8+ T cell epitopes as described previously [34]. T.O.D. = Time of Death. (B) The longitudinal sequence of SIVmac239 Gag197-211 QA15 from animal r04072 with position 205 highlighted in grey. (C) Direct ex vivo analysis of the ability of CD4+ T cells from r04072 to degranulate (as measured by CD107a) in response to whole AT-2-inactivated SIVmac239 or the Gag197-211QA15 peptide. Data is representative of two independent single replicate experiments performed with PBMC samples from 100 WPI. [file 1742-4690-9-91-S4.pdf]

Supplemental Figure 5

A

| Animal | MHC-II molecules present |
|--------|--------------------------|
| r95071 | DRB1*06                  |
| r95096 | DRB1*06, DRB1*10:07      |
| rhAJ11 | DRB1*03:06               |
| r00078 | DRB1*03:06               |
| r01064 | DRB1*03:06               |
| r98016 | DRB1*03:06               |

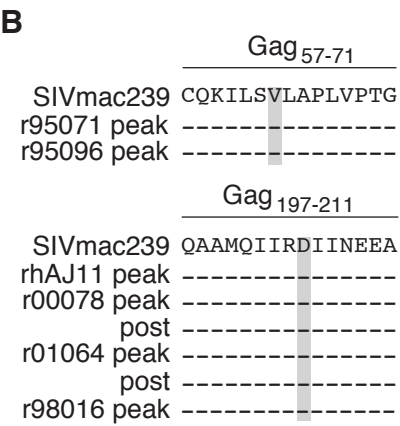

Supplement: Additional file 5 — Figure S5. CD8-depleted SIV ECs show no evidence of CD4+ T cell mediated escape within two highly targeted Gag CD4+ T cell epitopes. (A) SIV ECs from a previously described CD8+ cell depletion experiment [11,37] are listed along with their MHC-II molecules known to target Gag57-71 CG15 and Gag197-211 QA15. (B) Sequence of Gag57-71 CG15 and Gag197-211 QA15 at 14 days (peak) and >28 days (post) post experimental CD8 depletion. No variation is observed within these two regions of Gag. [file 1742-4690-9-91-S5.pdf]
